# The known and unknown about attention deficit hyperactivity disorder (ADHD) genetics: a special emphasis on Arab population

**DOI:** 10.3389/fgene.2024.1405453

**Published:** 2024-08-06

**Authors:** Nahed N. Mahrous, Amirah Albaqami, Rimah A. Saleem, Basmah Khoja, Mohammed I. Khan, Yousef M. Hawsawi

**Affiliations:** ^1^ Department of Biological Sciences, College of Science, University of Hafr Al-Batin, Hafr Al- Batin, Saudi Arabia; ^2^ Department of Clinical Laboratory Sciences, Turbah University College, Taif University, Taif, Saudi Arabia; ^3^ Department of Biochemistry and Molecular Medicine, College of Medicine, Al-Faisal University, Riyadh, Saudi Arabia; ^4^ Research Center, King Faisal Specialist Hospital and Research Center, Jeddah, Saudi Arabia

**Keywords:** ADHD, ASD, consanguinity, genetic variants, neurodevelopmental disorders, learning disorder

## Abstract

Attention deficit hyperactivity disorder (ADHD) is a clinically and genetically heterogeneous neurodevelopmental syndrome characterized by behavioral appearances such as impulsivity, inattention, and hyperactivity. The prevalence of ADHD is high in childhood when compared to adults. ADHD has been significantly advanced by genetic research over the past 25 years. However, it is logically conceivable that both genetic and/or non-genetic factors, such as postnatal environmental and social influences, are associated with ADHD phenotype in Arab populations. While genetic influences are strongly linked with the etiology of ADHD, it remains obscure how consanguinity which is an underlying factor for many genetic diseases, contributes to ADHD subtypes. Arabian Gulf Nations have one the highest rates of consanguineous marriages, and consanguinity plays an important contributing factor in many genetic diseases that exist in higher percentages in Arabian Gulf Nations. Therefore, the current review aims to shed light on the genetic variants associated with ADHD subtypes in Arabian Gulf nations and Saudi Arabia in particular. It also focuses on the symptoms and the diagnosis of ADHD before turning to the neuropsychological pathways and subgroups of ADHD. The impact of a consanguinity-based understanding of the ADHD subtype will help to understand the genetic variability of the Arabian Gulf population in comparison with the other parts of the world and will provide novel information to develop new avenues for future research in ADHD.

## 1 Introduction

Attention-deficit hyperactivity disorder (ADHD) is a common clinically and genetically transmitted neurological condition that affects an individual’s behavior (American Academy of Pediatrics, 2001). Symptoms of ADHD such as mood and anxiety disorders, conduct disorder, and oppositional defiant disorder are usually noticed at an early age of childhood and are increasingly recognized in adulthood (Biederman et al., 2006). It has been reported that among 3%–10% of those diagnosed with ADHD disorder in their childhood, and 6% of the overall population continue to develop noticeable ADHD conditions (Wender et al., 2001). The concept of ADHD as a clinical condition has changed over the 20th century until the onset of the twin and family studies (Foreman, 2006). The historical descriptions associated with ADHD ranged from early brain damage “neurodevelopmental disorders” to the indirect contribution of genetic and environmental factors (Sonuga-Barke et al., 2023). Nevertheless, many descriptions are consistent with the modern diagnostic strategies for ADHD.

In 1798, a Scottish physician named Sir Alexander Crichton was the earliest to reveal that the incapacity of attending may be innate or due to unnatural nervous sensibility (Lange et al., 2010). Crichton saw numerous examples of insanity in his therapeutic practice and had a growing interest in mental illness. Fidgety Philip (Heinrich Hoffmann), a German physician, in 1840s was the first to write allegory stories for children with ADHD (American Academy of Pediatrics, 2001). In the 1990s century, the description of the characteristic features of an individual with ADHD became the focus of several scientists (Thome and Jacobs, 2004). Geroge Still, a British pediatrician, in 1902 described an abnormal defect of moral control in children and found that some could not control their behavior in a typical way, but were still intelligent (Lange et al., 2010). In 1919, ADHD was blamed on brain damage, and numerous words were associated with the disorder, such as impulsive, inattentive, hyperactive, and short attention span (Burd and Kerbeshian, 1988). Medications for symptoms were discovered during the 1930s, and research on ADHD in both children and adults continued throughout the 19th and 20th centuries as a worldwide interest (Abdelnour et al., 2022).

Attention deficit hyperactivity disorder accounts for over 5% of frequent disorders within child and adolescent psychiatry (Drechsler et al., 2020). Physicians still struggle with diagnosis and treatment despite the enormous volume of research, more than 20,000 publications have been mentioned in PubMed during the past 10 years. Due to its heterogeneous nature, ADHD can present in opposite forms with frequent and variable comorbidities (Wahlstedt et al., 2009). Additionally, there is an overlap between the symptoms of ADHD and other diseases, such as autism spectrum disorder (ASD), major depressive disorder, bipolar disorder, and schizophrenia, which may or may not show up on a clinical assessment (Morimoto et al., 2021). Biomarkers or other objective criteria that could result in an automatic algorithm for the reliable identification of ADHD in an individual within clinical practice are currently absent, despite the fact that the disorder’s neurological and genetic foundations are undeniable.

## 2 Symptoms of ADHD

**FIGURE 1:**
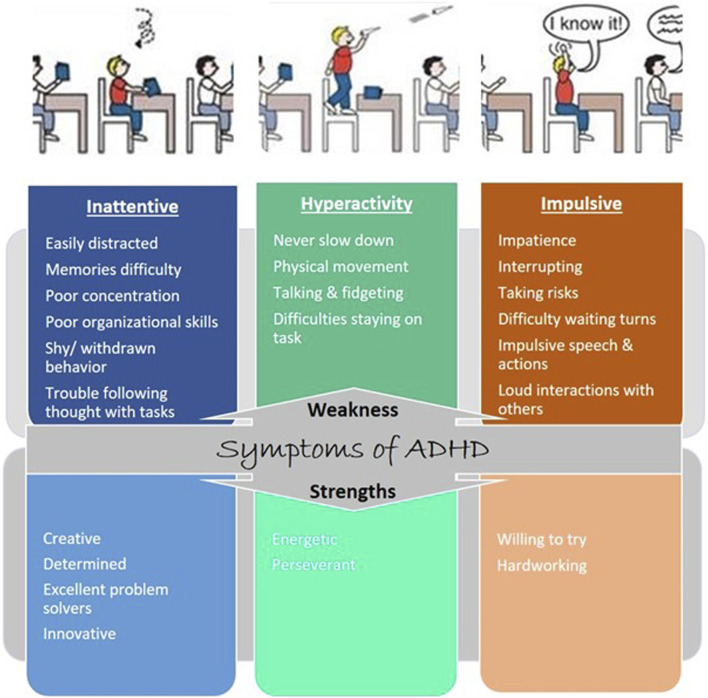
Symptoms of ADHD: weakness and strength. Each type of ADHD is tied to one or more characteristics: inattention and hyperactive-impulsive behavior.

The characteristic features of individuals with ADHD include hyperactivity, inattentiveness, and impulsiveness, which lead to impairments in daily living (Figure 1). Characterization of ADHD based on features is ensured by looking at a lack of moral control through inattentive and hyperactive-impulsive symptoms or a mix of both symptoms (Muller et al., 2011). Inattentive symptoms include trouble focusing, finishing tasks, following instructions, memories not always easy to recall, careless mistakes, and losing belongings (Muller et al., 2011). The hyperactive-impulsive symptoms include excessive talking and physical movement, interrupting conversations, answering before the question has been finished, dangerous behavior, choosing a smaller reward now rather than a larger reward later, finding boredom intolerable, and looking for stimulation. Some people have combined symptoms of both inattentive and hyperactive-impulsive symptoms (American Psychiatric Association, 1987).

**FIGURE 2:**
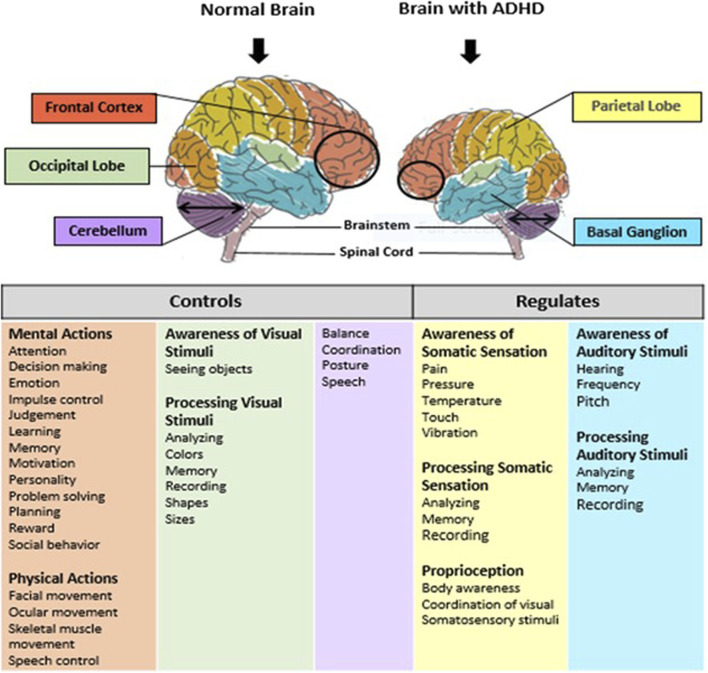
Schematic representation of normal brain and brain with ADHD. Brain consists of five main regions: prefrontal cortex, occipital lobe, cerebellum, the basal ganglia, and parietal lobe. These regions control and regulate several functions such as: inhibition, memory, planning and organization, motivation, processing speed, inattention and impulsivity. Brain development in individual with ADHD is smaller in the prefrontal cortex, and cerebellum regions as compare to a normal brain. Also, ADHD has been linked to deficits in the functioning of the prefrontal cortex, the basal ganglia, cerebellum and parietal lobe.

**FIGURE 3:**
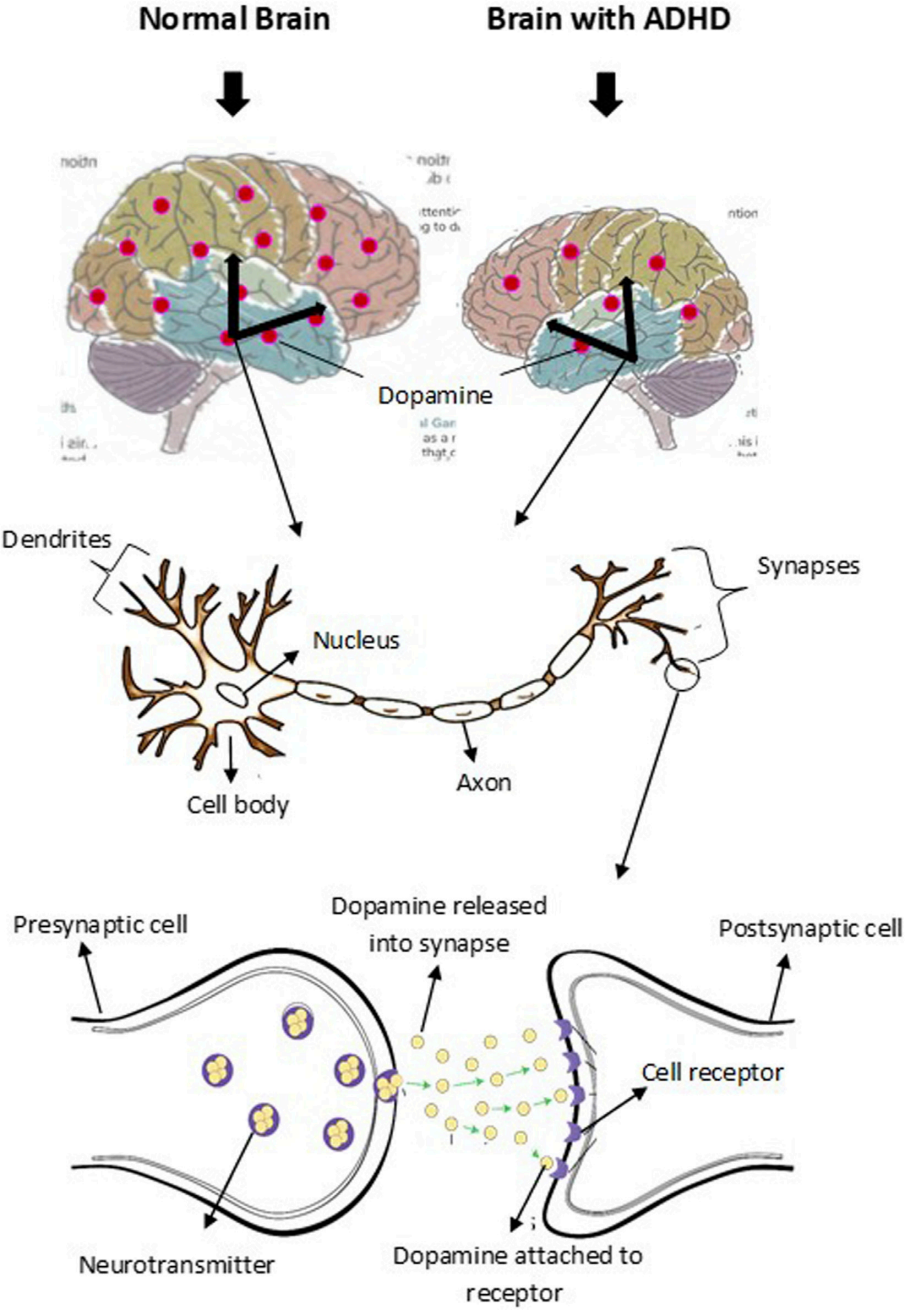
Schematic representation of dopamine level and transmission in normal brain and brain with ADHD. Dopaminergic neuron is located in the midbrain and control various functions. The level of dopamine in normal brain is higher than the level in brain with ADHD. Dopamine level contributes to the symptoms of inattention, hyperactivity, and impulsiveness in ADHD.

Brain development and functions are achieved by expressing multiple genes distributed in four brain regions: frontal cortex, cerebellum, parietal lobe, and basal ganglion (Table 1). These genes are essential in regulating systemic sympathetic activity and contribute to the behaviors associated with dopamine levels. Normal brains and brains with ADHD develop in the same manner. However, the brain with ADHD develops more slowly overall, particularly in the frontal lobes that help with impulse control and concentration (Figure 2). As a result, neurodevelopmental disorders including ADHD may not mature to the same degree as a normal brain, though, depending on how severe the symptoms are (Gehricke et al., 2017). In fact, people with childhood ADHD diagnoses had a smaller overall brain capacity than adults without such a diagnosis, according to neuroscientists (Castellanos et al., 2002). In the areas of their brains impacted by ADHD, there was greater cortical thinning and less cortical thickness in the outer layer (Konrad et al., 2012). This essentially indicates that there were fewer brain cells in these regions in these people. Normal brains may produce brain cells more quickly than brains with ADHD, which could be one explanation for this. Therefore, before cortical thinning begins, those people have a higher initial level of grey matter. In addition, the brain with ADHD has a lower level of dopamine, which helps control the brain’s reward and regulates movement and emotional responses, in comparison with the normal brain (Figure 3). As a result, the ADHD brain cannot stay motivated and experiences functional changes and weak connectivity. In general, the structure, chemistry, and function of the ADHD brain differ from those of the non-ADHD brain.

**TABLE 1:** A representation of a collective of genes that encode for primary altered structural and functional brain changes within the five brain regions that were found to be correlated with the mechanism of actions found in attention-deficit hyperactivity disorder (ADHD).

Risk Genes	Gene Function	Frontal Cortex	Occipital	Cerebellum	Parietal Lobe	Basal Ganglion
ADRA2A	Alpha-2A adrenergic receptor, regulates systemic sympathetic activity and cardiovascular responses.	√				
COMT	Catechol-O-methyltransferase, involves in the inactivation of the catecholamine neurotransmitters (dopamine, epinephrine, and norepinephrine).			√		√
DRD1	Dopamine receptor D1, regulates the memory, learning, and the growth of neurons and mediates some behaviors.	√		√	√	√
DRD4	Dopamine receptor D4, regulates the dopaminergic system and contributes to the behaviors connected with dopamine levels.	√		√	√	√
HTR1B	Encodes the 5-hydroxytryptamine receptor 1B, its function differs upon its brain location.	√				
MAOA	Monoamine oxidase A, regulator for normal brain function.	√			√	
NET1	Neuroepithelial cell-transforming gene 1, regulate cell signaling.	√			√	
NR3C1	Glucocorticoid receptor, regulates genes controlling the development, metabolism and immune response.			√		
SLC6A3	Encodes sodium-dependent dopamine transporter, provides instructions for making dopamine transporter.	√			√	√
SLC6A4	Encodes 5-HTTLPR, the sodium-dependent serotonin transporter, responsible for serotonin reuptake into the presynaptic neuron.	√		√		√

## 3 Diagnosis of ADHD

**FIGURE 4:**
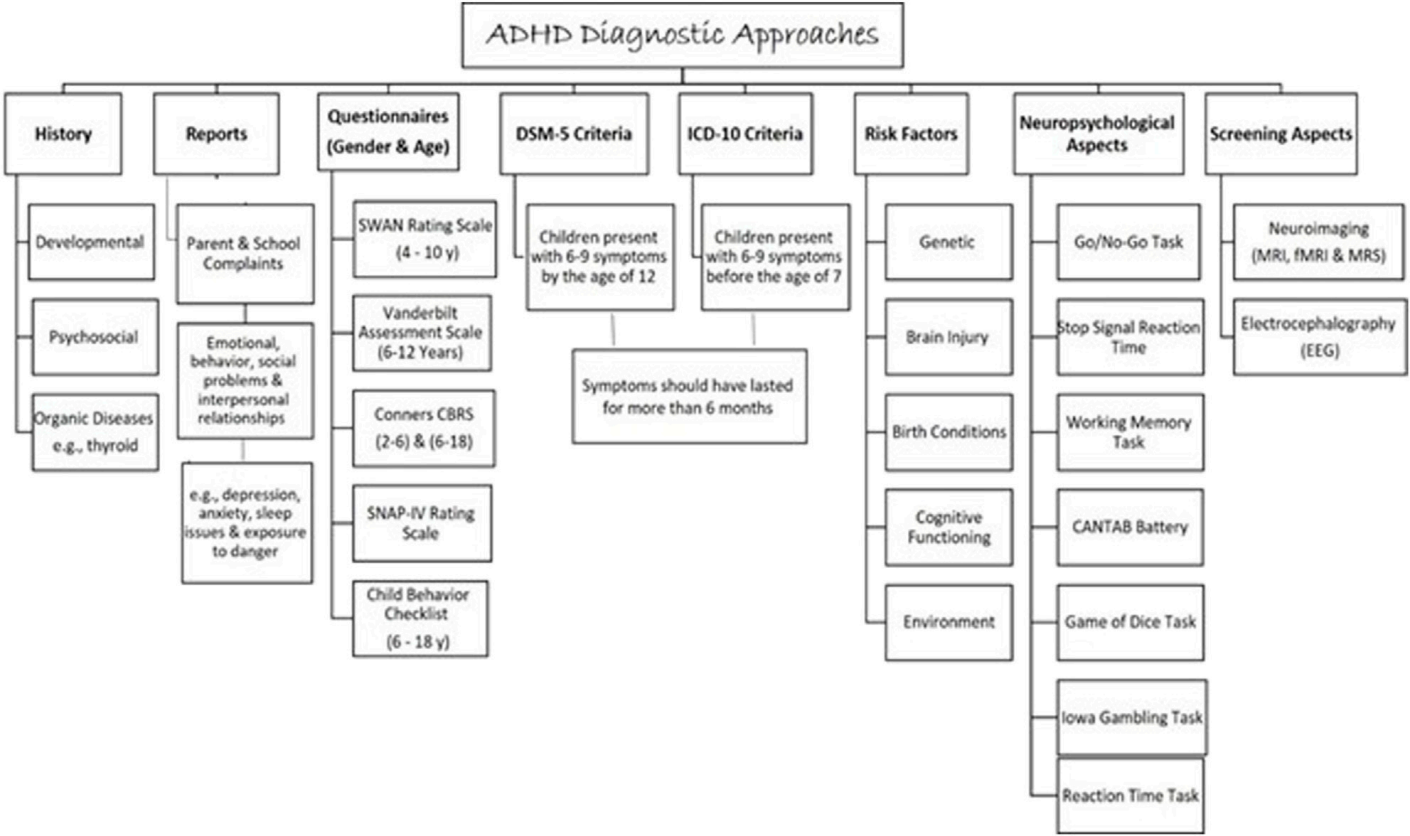
An approach to the evaluation and the diagnosis of ADHD in children and adults.

Attention deficit hyperactivity disorder is considered a major public health problem (Ferguson, 2000) as it causes serious impairment at school, home, and society (American Psychiatric Association, 1987). Therefore, to diagnose ADHD, impairment in school performance and daily life is assessed by using a symptoms inventory questionnaire for ADHD described in the Diagnostic and Statistical Manual of Mental Disorders (DSM-5) or the International Statistical Classification of Diseases and Related Health Problems (ICD-10) (Bell, 1994; Organization, 2004) (Figure 4). Children diagnosed with ADHD presented with 6–9 symptoms by the age of 12 for (DSM-5) or before the age of 7 for (ICD-10). Symptoms should have lasted for more than 6 months (Bell, 1994). The assessment must be carried out by a psychiatrist, pediatrician, or ADHD specialist. A full developmental and psychosocial history, and physical examination, alongside family and school reports, are essential to diagnose ADHD (Organization, 2004).

Current available ADHD objective assessment includes neuropsychological tests, neuroimaging, and electroencephalography (EEG). Research has shown that individuals with ADHD exhibit distinct genetic anomalies compared to those without the disorder. Unfortunately, there is insufficient evidence on the use of these tests to establish ADHD diagnosis (Kemper et al., 2018). Besides, there is no genetic testing in use to diagnose ADHD regardless of its high heritability (approximately 80%) (Thapar, 2018). However, numerous studies have utilized different types of genetic testing to understand and explore the genetic involvement in ADHD. Gene-wide association studies (GWAS) of single nucleotide polymorphisms (SNPs) and copy number variants (CNVs), next-generation sequencing (NGS), and single nucleotide variants (SNVs) for example, have illustrated the genetic contribution in the ADHD etiology (Hawi et al., 2015; Lima Lde et al., 2016; Grimm et al., 2018). The array of comparative genomic hybridization (aCGH) genetic testing has also helped identify the associated CNVs in many neuropsychiatric disorders including intellectual disability (ID), ASD, and ADHD (Baccarin et al., 2020). In this context, CNVs in multiple chromosomal regions have been linked with ADHD (5p13, 14q12, and 17p11) (Arcos-Burgos et al., 2004; Ogdie et al., 2006). In addition, Demontis et al. (2019) have shown many genetic variants correlated with ADHD on chromosomes 1–5, 7–8, 10, 12, and 15–16 (Demontis et al., 2019).

Given that, it is important to know that ADHD symptoms can overlap with other coexisting conditions, such as the development of substance use, motor control, conduct, language, learning, and mood disorders in children and bipolar, obsessive-compulsive, and personality disorders in adults (Bonati et al., 2018). While severe attention, hyperactivity, and impulsive impairments are hallmarks of ADHD, the disorder sometimes coexists with other neurological conditions, including ASD (Dougnon and Matsui, 2022). Along with repetitive and limited behavior and interests, ASD is linked to poor communication and social interaction abilities. Therefore, differentiation between these disorders during diagnosis is required.

Attention-deficit hyperactivity disorder is associated with a variety of neuropsychological (NP) characteristics and underlying neurobiological pathways (Curatolo et al., 2010). An early dual pathway model distinguished between an inhibitory/executive function pathway and a motivational/delay aversion pathway (also referred to as the “cool” and “hot” executive function pathways in later publications). These pathways are related to different neurobiological networks. Twenty-five years ago, ADHD was diagnosed as a disorder of inhibitory self-control (Castellanos and Tannock, 2002; Castellanos et al., 2006). Although other routes, such as time processing, have been introduced, it is challenging to pinpoint the exact number of pathways that could exist. For example, Coghill and colleagues (2014) distinguished six cognitive characteristics in children with ADHD, including working memory, inhibition, delay aversion, decision-making, timing, and response time variability, using data from seven subtests of the computerized Cambridge NP test battery (Coghill et al., 2014). Other empirical research have distinguished between three (Gomez et al., 2014) or four (Fair et al., 2012) NP profile groups, which all included children with ADHD as well as typically developing (TD) children, differing in severity but not in the type of profile. These studies used latent profile or cluster analysis of NP tasks in large ADHD samples. However, in the process of looking for subgroups, some additional empirical research found performance profiles that were specific to ADHD (poor cognitive control (van Hulst et al., 2015), with attentional lapses and fast processing speed (Bergwerff et al., 2019), among other profiles that were shared with TD controls). Divergent subgrouping outcomes can, of course, also be attributed to different compilations of domains that have been studied, which limits the comparability of these investigations.

### 3.1 Animal models of ADHD

Characteristics of ADHD are based upon the presence of three main criteria: attention, hyperactivity, and impulsiveness, which must be present before the age of 7. Identifying biological markers to describe the core disorder of ADHD may help to develop new diagnostic and treatment strategies. Several animal models of ADHD have been proposed to provide insight for this purpose although they cannot accurately reflect human psychiatric condition (Sontag et al., 2010; Russell, 2011).

Animal models should meet the necessary validation criteria: 1) face validity, which includes the three core symptoms of ADHD, etiology, treatment, and physiological basis of the modeled disease; 2) construct validity, which conforms to pathophysiological mechanisms; and 3) predictive validity, which is the ability to predict unknown aspects of the ADHD (Viggiano et al., 2004; Sontag et al., 2010; Russell, 2011). Various animal models with distinctly different neural defects (rats, mice, and monkeys) have been proposed to reflect the heterogeneous nature of ADHD, yet none can ensure which model best represents neither the disorder nor its subtypes. These models can be divided into: 1) genetic/trans-genetic, and 2) pharmacological animal models (Table 2) (Kim et al., 2024).

**TABLE 2:** Propose animal models in attention-deficit hyperactivity disorder (ADHD) research (Regan et al., 2022; Kim et al., 2024).

Genetic/Trans-genetic animal models	Pharmacological animal models
Spontaneously hypertensive rat (SHR) • Inbred strain derived from WKY rat • Hyperactivity, impulsivity and inattention	Neonatal 6-hydroxydopamine rats
Naples high-excitability rats • Lower expression of DAD1 transcripts in NHE • 26 mRNAs expressed in the prefrontal cortex • Hyperactivity and inattention	Neonatal hypoxia in rats
Acallosal mice • Hyperactivity and impulsivity	Developmental cerebellar stunting in rats
	Metylazoxymethanol (MAM)
	Amphetamine
	Alpha-difluoromethylornithine (DFMO)
	Dexamethasone
Alpha synuclein lacking mice • Lack of alpha or/and gamma synuclein • Hyperactivity	Maternally stressed mice
Dopamine-transporter knockout mouse (DAT-KO) • Mouse lacks the DA transporter (DAT) gene	
Coloboma mutant mouse • Disruption in about 20 genes (e.g., SNAP) • Hyperactivity, impulsivity and inattention	
Thyroid hormone receptor (TR)-beta (1) transgenic mouse • Mouse carries a mutant human TRβ1 gene, which was derived from a patient diagnosed to suffer from a resistance to thyroid hormone (Buitelaar et al., 2022) syndrome • Hyperactivity, impulsivity and inattention	

Of all the rodent models, research demonstrated that spontaneously hypertensive rat (SHR), a genetic model derived from Wistar-Kyoto (WKY) rats, fulfills many of the validation criteria for ADHD. Nevertheless, it shows several characteristic features of the disorder including inattention, impulsivity, and hyperactivity when compared to normotensive, WKY rats from various tests (Regan et al., 2022). Additionally, SHR shows neuropathological changes similar to those observed in patients with ADHD when compared with the WKY (e.g., smaller prefrontal cortex and hippocampus and fewer neurons). Several studies have reported developmental changes in the expression of dopamine transporter gene (DAT) in specific regions of the brain and dopamine uptake and utilization (Cook et al., 1995; Kirley et al., 2003; Bobb et al., 2005). Decreased expression of DAT4 in the prefrontal cortex and increased density of DAT1 and DAT5 in the neostriatum were observed in young male SHR (Li et al., 2007). However, transgenic animals such as Naples high excitability rat (Kim et al., 2020) performers promise to be useful models for investigating novel genes associated with ADHD *in vivo*. Other proposed animal models focus on the less important symptom of hyperactivity and may offer an opportunity to explore the role of dopamine in brain illness and assess new ADHD treatments.

## 4 Genetics and heritability of ADHD

Early molecular genetic research on ADHD aimed to link the disorder with genes that had prior plausibility in its development. Given that medications addressing ADHD primarily act on dopaminergic or noradrenergic transmission, many studies focused on investigating “candidate genes” within these pathways. Variants in genes related to neurotransmitter regulation, such as dopamine (Del Campo et al., 2011) and norepinephrine (Arnsten and Pliszka, 2011) pathways, appear to play a significant role. Furthermore, understanding the gene functions associated with altered brain function in neurodevelopmental disorders can unravel the biological pathways involved in ADHD.

The heritability of ADHD is between 75% and 90% based on twin and family studies. Recent studies have identified a strong hereditary component in ADHD, suggesting that genetics may account for up to 70%–80% of the variance in ADHD risk (Thapar, 2018). Multiple genes are implicated, each exerting a modest effect (Faraone and Larsson, 2019). Furthermore, it was discovered that the heritability of the inattentive and hyperactive-impulsive components of ADHD was comparable in males and females (Faraone and Larsson, 2019). Understanding the genetic underpinnings of ADHD is a complex and evolving field of research. This review delves into the intricate web of genetic factors that contribute to the development and manifestation of ADHD, shedding light on the interplay between genes and this neurodevelopmental disorder. It has recently been demonstrated that there are heritable components linked to ADHD in the structural and functional brain connections evaluated in families with children diagnosed with ADHD (Sudre et al., 2017). In a similar vein, it was discovered that there was a high heritability of the event-related potentials (ERPs) generated in a Go/No-Go test that measured reaction inhibition, which is known to be changed in ADHD (Anokhin et al., 2017).

Twin and family studies provide valuable insights into the genetic basis of traits and diseases. The key idea in twin studies is to compare the within-pair similarities of Monozygotic (MZ) twins to those of Dizygotic twins (DZ). If a trait or characteristic has a stronger correlation within MZ pairs compared to DZ pairs, it suggests a significant genetic influence on that trait. On the other hand, if the resemblance is similar between MZ and DZ pairs, it implies a greater role for environmental factors. Such studies also help researchers understand the mode of inheritance, estimate heritability, identify genetic markers, and unravel the interplay between genes and the environment. Furthermore, information gathered from family and twin studies is often used to design and interpret GWAS, which aims to identify specific genes associated with diseases.

A study with 894 individuals diagnosed with ADHD and 1,135 of their siblings, aged 5–17 years, uncovered a nine-fold increase in the likelihood of ADHD among the siblings of individuals with ADHD when compared to siblings in the control group (Chen et al., 2008). Another twin study conducted by Larsson et al. (2012) applied twin methods to investigate the extent to which ADHD is etiologically distinct from subthreshold variations in ADHD symptoms. Interestingly, when the extreme and subthreshold continuous ADHD characteristic symptoms were evaluated in the Swedish twins, a significant hereditary component was also discovered (Larsson et al., 2012). Family and twin studies have also contributed immensely in identifying and verifying genes that are associated with ADHD. A study by Lasky-Su et al. (2008) investigated 958 ADHD proband-parent trios by genotyping over 600,000 SNPs (Kim et al., 2020). Two novel variants were identified and are located in intronic regions of genes CDH13 and GFOD1. The CDH13 gene provides instructions for producing a calcium-dependent cell-cell adhesion protein crucial for influencing synaptic plasticity and neuronal development. Various genome-wide genetic methods, such as linkage analyses, association studies, and investigations into copy number variations, have identified connections between the CDH13 gene and diverse neurodevelopmental disorders. This suggests that alterations in CDH13 may contribute to the susceptibility or development of conditions impacting the nervous system during early development (Rivero et al., 2015). Another gene that was found to be significantly associated with the criteria is SLC9A9 (Lasky-Su et al., 2008). SLC9A9 is a gene of interest and has also been found to be implicated in other ADHD studies (Zhang-James et al., 2011; Zhang-James et al., 2012). The outcome of such studies underscores a significant genetic impact on the development of ADHD. In essence, the observed heightened risk in siblings of individuals with ADHD highlights the considerable influence of genetic factors in the onset of this neurodevelopmental disorder. This insight enhances our understanding of the complex interplay between genetics and environmental factors in the susceptibility to ADHD.

Genome-wide association studies have been instrumental in identifying specific genetic markers associated with ADHD (Franke et al., 2009). These markers are linked to the disorder and offer insights into related traits and comorbidities (Mehta et al., 2020). The intricate genetic landscape of ADHD involves a complex interplay of risk and protective factors, environmental influences, and gene-environment interactions (Kim et al., 2020). Moreover, epigenetic mechanisms contribute to the dynamic nature of ADHD genetics (Mill and Petronis, 2008). Environmental factors, such as prenatal exposure to certain substances or stressors, can modify gene expression, potentially influencing ADHD susceptibility (Mill and Petronis, 2008). Understanding these epigenetic processes is crucial for a more nuanced comprehension of the disorder’s etiology (Nigg, 2018).

In a genome-wide association (GWA) research meta-analysis of ADHD, Demontis et al. (2023) included 186,843 controls and 38,691 ADHD sufferers. About 27 genome-wide important loci were found, and 76 possible risk genes that are enriched in genes expressed specifically in the early stages of brain development were highlighted. In general, several brain-specific neuronal subtypes and midbrain dopaminergic neurons were linked to the hereditary risk of ADHD (Demontis et al., 2023). GWAS have led to the conclusion that there is an overlap in the genetics of ADHD and Bipolar disorder. For instance, DAT1, recently renamed as SLCA3 (Villani et al., 2018), is hypothesized to be associated with ADHD and has been confirmed to be linked to bipolar disorder. Interestingly, in psychiatry, genetic markers at the DAT1 locus appear to be able to predict clinical heterogeneity since the non-conduct disordered subtype of ADHD is associated with DAT1. In contrast, other subgroups do not appear to be connected. It is becoming increasingly apparent that the candidate genes for bipolar mania and candidate genes for an ADHD subtype are related (Polanczyk et al., 2014). In a study conducted in Oman, researchers investigated the frequency of VNTR alleles in DAT1 and their correlation with ADHD. They genotyped 92 Omani children with ADHD and 110 healthy controls, identifying two common alleles (DAT19 and DAT110). The DAT1*10 allele showed similar distribution between ADHD (64.6%) and control groups (60.9%), but was more prevalent in ADHD males (69.4%) compared to females (55%), a distinction absent in controls (Simsek et al., 2005). Given the high rate of consanguineous marriages in the GCC region, further twin studies and broader research are warranted to explore the genetic foundations of disorders like ADHD in the Arab world. The discovery of novel genetic variants associated with ADHD will foster the early detection of the disease.

Furthermore, epigenetic processes, which regulate gene expression, are proposed as a significant biological marker and potentially a molecular mediator of genetic and environmental influences on ADHD. A high level of DNA methylation has been associated with repression of gene expression, but recent data have shown that this association can vary depending on factors including the location of the methylation in the gene sequence. Several studies have concluded that investigating epigenetic effects on human behavior is challenging because epigenetic changes vary widely from one tissue to another. In the brain alone, DNA methylation can vary considerably from one region to another or from one system to another. However, there are also cross-tissue correlation patterns of individual-specific epigenetic marks, suggesting that DNA methylation studies of peripheral tissue DNA in psychiatry are generally considered informative (Wilmot et al., 2016; Mirkovic et al., 2020).

Vasoactive intestinal peptide receptor 2″encoded by VIPR2″ is present throughout the central nervous system and peripheral tissues. In a methylome-wide exploration of DNA methylation in peripheral tissue Wilmot et al. (2016) examined salivary DNA from children with ADHD compared to non-ADHD controls (Wilmot et al., 2016). They have found that probes in VIPR2 showed decreased CpG methylation in the saliva of children with ADHD. CpG methylation, which is essential for regulating gene expression, impacts a wide range of biological processes, from development to aging and inflammation. CpG islands, located in DNA methylation regions within promoters, are known to control gene expression by silencing transcription, playing a vital role in gene expression and tissue-specific functions (Wilmot et al., 2016).

Mutations in the ST3GAL3 gene, which encodes the Golgi enzyme β-galactoside-α2,3-sialyltransferase-III, are believed to play a role in the development of advanced cognitive abilities. This enzyme is crucial for the addition of sialic acid to glycoproteins and glycolipids, a process essential for proper brain function. Disruptions in this enzyme’s activity due to mutations in ST3GAL3 could potentially affect neural connectivity and signaling, thereby influencing higher-order cognitive functions such as learning, memory, and problem-solving. Research into these mutations helps to understand how specific genetic factors contribute to the complexities of cognitive development (Mijailovic et al., 2022). A genome-wide methylome analysis uncovered that ST3GAL3 undergoes differential methylation throughout the progression of ADHD. This suggests that gene-environment interactions might influence the expression of ST3GAL3, affecting the epigenetic regulation of brain development and maturation (Walton et al., 2017). Rivero et al. (2021) has established when investigating on mice models, that male St3gal3 heterozygous mice exhibit cognitive deficits, while female heterozygous mice show increased activity and improved cognitive control compared to the wildtype mice (Rivero et al., 2021). The research also identified subtle, sex-specific, and region-specific changes in the expression of various markers associated with oligodendrogenesis, myelin formation, protein sialylation, and cell adhesion/synaptic target glycoproteins of ST3GAL3. These findings suggest that ST3GAL3 haploinsufficiency leads to sex-dependent alterations in cognition, behavior, and brain plasticity markers. Additionally, St3gal3-deficient mice displayed behavioral changes similar to those observed in ADHD, such as differences in locomotor activity and cognitive performance, with male heterozygous mice specifically showing memory impairments in place learning tasks, a common issue in ADHD (Rivero et al., 2021).

Despite significant strides, challenges persist in unraveling the complete genetic architecture of ADHD. Heterogeneity within the disorder (Faraone, 2000), the influence of non-genetic factors (Mill and Petronis, 2008), and the intricate nature of gene interactions (Acosta et al., 2011) pose ongoing research challenges. In the realm of personalized medicine, genetic insights into ADHD hold promise for tailoring interventions (Buitelaar et al., 2022). Identifying genetic markers may pave the way for targeted therapies, allowing for more precise and effective treatment approaches (Mehta et al., 2020).

In the context of GWAS, there are only a few studies that directly identified variants with ADHD. The absence of positive findings suggests that the influence of individual common ADHD risk variants is likely to be minor, given the disorder’s high heritability. Alternatively, this prompts consideration that other types of variants, such as rare ones, might play a more significant role in contributing to the heritability of ADHD. These findings suggest that more GWAS should be performed from countries like Saudi Arabia with high consanguinity rates to aid researchers in identifying rare variants that are associated with ADHD.

Differences in linkage disequilibrium (LD) patterns among populations play a pivotal role in influencing the transferability of GWA findings. A variant strongly associated with a trait in one population may not exhibit a detectable association in another population due to differences in LD with the unknown causal variant. Given the pronounced disparities in LD observed among European, East Asian, and Arab populations, it becomes essential to account for these population-specific LD variations when evaluating the transferability of GWA-identified associations.

Research on ADHD in the Arab world, especially in the GCC region, has primarily concentrated on behavioral and epidemiological studies, with limited attention to the genetic and epigenetic factors underlying the disorder. The Arab population, characterized by large family sizes, high rates of consanguinity, and frequent first-cousin marriages, presents a unique genetic landscape that leads to increased homozygosity for both monogenic and polygenic diseases, and the accumulation of deleterious recessive alleles. This distinctive genetic profile makes the Arab population particularly valuable for genetic and epigenetic research, including twin and extended family studies, which could offer profound insights into the complex interplay between genetics and health. Despite these opportunities, the representation of the Arab population in global GWAS is inadequate, and the applicability of GWA findings to this group remains underexplored. Expanding genetic and epigenetic research on ADHD in the GCC is crucial to ensure that findings from global studies are relevant and beneficial to the Arab population, and to enhance our understanding of the disorder’s biological foundations in this unique context.

## 5 Epidemiology and prevalence of ADHD

Although prevalence rates and reported changes in the prevalence of ADHD vary greatly depending on country and region, technique, and sample, it appears to be a global phenomenon (Polanczyk et al., 2015). In 2014, Polanczyk et al. conducted a meta-analysis study that revealed a 5.8% global prevalence rate for children and adolescents (Polanczyk et al., 2014). Different criteria for identifying ADHD are causing slightly higher (e.g., 7.2%) prevalence percentages reported by other meta-analyses conducted by Thomas et al. (2015). Peak prevalence may be significantly greater in some age groups, such as 13% in 9-year-old boys although prevalence statistics in children and adolescents represent average values across the entire age range (Bachmann et al., 2017). The estimated prevalence of ADHD in adults worldwide is 2.8% with greater rates observed in high-income nations (3.6%) compared to low-income ones (1.4%) (Bachmann et al., 2017).

### 5.1 Current status of ADHD in Arabian Gulf Nations

The six Arab Gulf states that make up the Gulf Cooperation Council (GCC) consist of Bahrain, Kuwait, Oman, Qatar, the United Arab Emirates, and Saudi Arabia, which is the largest country, and are all disproportionately affected by the “youth bulge” with a combined population of approximately 54 million (Thygesen et al., 2021). However, the GCC is classified as in the “second phase of demography in transition” (Omran, 2005). As a result, this stage is frequently distinguished by high rates of conception and births and the prevalence of a population structure resembling a pyramid-like population structure. Systematic clinical data in this regard point to a significant number of children with disabilities in the GCC (Chan et al., 2021). This might be caused by the high rates of polygamous marriages and consanguinity among married couples, especially among older males, as well as high fertility and frequent childbirth. Moreover, the majority of GCC nations have not yet created policies to assist young people with special needs (Qoronfleh et al., 2019). Although it is still in its early stages, research on the prevalence of ADHD diseases in the GCC has emerged (Farah et al., 2009). To fill this gap in the literature, this study aimed to critically review the literature on the prevalence rates of ADHD disorders in the GCC (Table 3). A glimpse into the literature shows that ADHD disorder has received some attention in this region. In a research of PubMed, a total of 33 studies from the six countries were found (Chan et al., 2021). Focusing on the genetics of ADHD, only eight studies were found in the KSA, one in UAE, Kuwait, Qatar, and Oman.

**TABLE 3:** Present studies conducted in the Gulf Cooperation Council (GCC) region. ADHD, attention-deficit hyperactivity disorder.

No.	Country	Study setting	Sample size	Age range (Years)	Screening tool	Disorder screened	Positive cases (%)	Author (Year) References
1	Saudi Arabia	Hospital	n = 416	0–18	DSM III-R/DSM IV	ADHD	ADHD = 106 (25.5%)	Al-Haidar (2003)
2		School	n = 1,287	6–13	ADDES	ADHD	ADHD = 572 (45.11%)	Al Hamed et al. (2008)
3			n = 642	7–9	DSM-IV-TR	ADHD	ADHD = 33 (5.0%) [DSM-IV-TR]	Alqahtani (2010)
4			n = 708	7–9	VADHDDRS	ADHD	ADHD = 29 (4.1%)	Jenahi et al. (2012)
5			n = 1,009	6–15	ADDES	ADHD	ADHD = 107 (11.3%)	Jenahi et al. (2012)
6			n = 929	6–12	VADHDDRS	ADHD	ADHD = 46 (11.6%)	AlZaben et al. (2018)
7			n = 2,770	6–12	ADHDS	ADHD	ADHD = 321 (11.6%)	Fatima and Ahmad (2018)
8	United Arab Emirates	School	n = 200	11–14	DSM-IV	ADHD	ADHD = 25 (12.5%)	Cook et al. (1995)
9	Kuwait	Hospital	n = 135	6–15	VADHDDRS-PRS (Arabic)/SSPI/WIS/MINI-KID	ADHD	ADHD = 70 (51.9%)	Nigg (2018)
10	Qatar	School	n = 4,489	6–19	SNAP-IV RS	ADHD	ADHD = 373 (8.3%)	Bradshaw and Kamal (2017)
11	Oman	School	n = 328	9–10	VADHDDRS-TAS	ADHD	ADHD = 28 (8.8%)	Al-Ghannami et al. (2018)

Research specifically focused on the genetics of ADHD within GCC populations is very limited. Most of the genetic studies on ADHD have been conducted in Western populations (Larsson et al., 2006; Thomas et al., 2015). However, genetic factors contributing to ADHD are expected to be similar across different populations due to the high heritability of the disorder (Faraone and Larsson, 2019). Generally, the GCC appears to be among the growing economies where the environmental aspect plays an important role in population health. In addition to genetic factors, cultural, social, and environmental factors in the GCC can influence the prevalence and presentation of ADHD (Zheng et al., 2020). For example, in 2022, a study by Setyanisa et al. presented those differences in educational systems, parenting styles, and societal attitudes toward mental health can impact the diagnosis and management of ADHD (Setyanisa et al., 2022). While the core genetic risk factors for ADHD are likely to be similar globally, there may be population-specific genetic variants in the GCC countries due to genetic drift, founder effects, and consanguinity, which is more common in the GCC than in many other regions specifically in Saudi Arabia (Bakry et al., 2023).

### 5.2 Consanguinity in Saudi Arabia

The Arabian Peninsula is known for its high rate of consanguinity. In Saudi Arabia, consanguineous marriage accounted for 52.0% of marriages, with an average inbreeding value of 0.0312 (Al-Abdulkareem and Ballal, 1998). Consanguineous marriage is one of the main risk factors that contribute to the spread of genetic syndrome. Therefore, there is a significant prevalence of genetic abnormalities in Saudi Arabia (Bakry et al., 2023). Several neurodevelopmental disorders were studied genetically and several variants were reported globally. However, Arab countries specifically Saudi Arabia need more attention to discover the genetic route of ADHD. In this land of the world, the genetic cause of ADHD is still a mystery due to the high rate of consanguinity.

In consanguineous populations such as Saudi Arabia, the probability of both parents carrying the same recessive gene mutation is significantly higher compared to low rates of consanguinity populations (Hamamy, 2012). Accordingly, the incidence of autosomal recessive disorders is more frequent in populations with a high rate of consanguinity (Khayat et al., 2024). High rates of consanguinity can increase the prevalence of certain genetic disorders, including those with complex inheritance patterns (Mahrous et al., 2023; Semlali et al., 2018a; Semlali et al., 2018b; Hawsawi et al., 2019), and ADHD. ADHD is a multifactorial disorder, meaning multiple genes and environmental factors influence it (Zayats and Neale, 2019). Certain genetic variants have been associated with an increased risk of ADHD, affecting neurotransmitter systems such as dopamine and serotonin (Yadav et al., 2021). Consanguineous marriages increase the probability of inheriting identical genetic variants from both parents. This can increase the risk of recessive genetic disorders and also contribute to the expression of complex genetic traits, including ADHD (Jackson et al., 2018). The increased genetic homogeneity in consanguineous populations can lead to a higher prevalence of certain alleles that may contribute to ADHD.

Studies in Saudi Arabia have identified a higher prevalence of certain genetic disorders due to high rates of consanguinity. Unfortunately, research specific to ADHD in consanguineous populations is limited but suggests a potential link between consanguinity and the genetic factors contributing to ADHD (Khayat et al., 2024). Therefore, the higher rates of ADHD in consanguineous populations could necessitate tailored public health strategies, including genetic counseling and targeted interventions. Understanding the genetic link can help in developing specific diagnostic tools and treatments for ADHD in populations with high consanguinity rates.

While the direct link between consanguinity and ADHD genetics in Saudi Arabia requires more research, the increased prevalence of genetic variants in consanguineous populations can contribute to a higher incidence of ADHD. This underscores the importance of genetic studies and targeted healthcare strategies in these populations (Alrefaei et al., 2022).

## 6 Current treatment and management of ADHD

**FIGURE 5:**
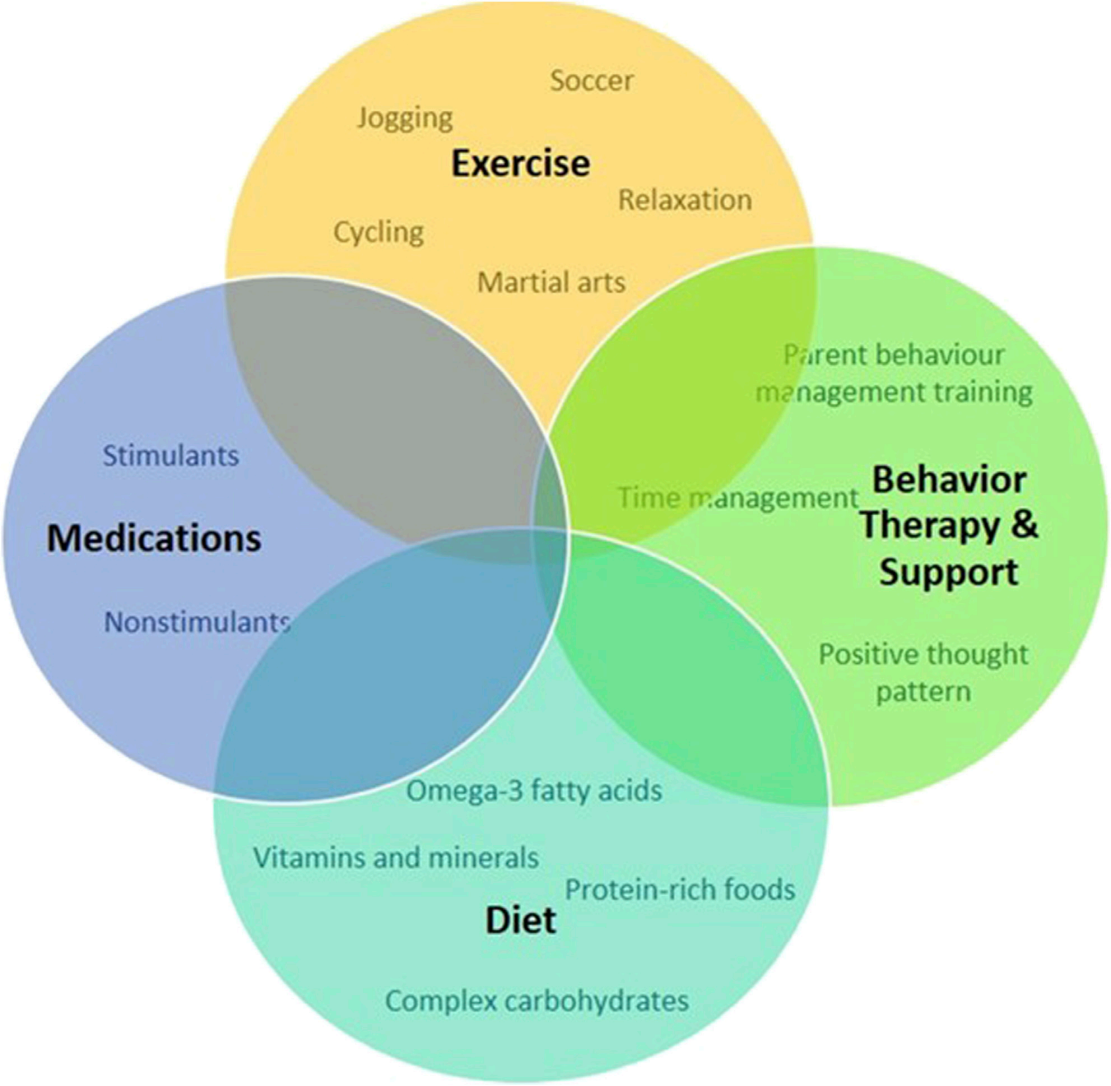
Schematic representation of approaches to treat and manage symptoms and promote positive behavior for individuals with ADHD.

Living with ADHD presents a unique set of challenges and opportunities. While the journey may be marked by moments of distraction and impulsivity (Wiklund et al., 2017), individuals with ADHD often possess remarkable creativity and an ability to think outside the box (White and Shah, 2011) (Figure 1). Attention deficit hyperactivity disorder is not a one-size-fits-all condition; it manifests differently in each person. Some may struggle with maintaining focus (Aliabadi et al., 2016), while others may grapple with impulsive decision-making (Hayashibara et al., 2023). However, it is crucial to recognize that ADHD is not a deficit of attention but a difference in attention (Baas et al., 2008). Embracing this perspective allows individuals to tap into their strengths and cultivate coping mechanisms (Fredrickson, 2000). One of the keys to navigating life with ADHD is developing personalized strategies (Lazar and Stein, 2017) (Figure 5). From utilizing visual aids and setting clear routines to breaking tasks into manageable chunks, individuals with ADHD can enhance their productivity and overall wellbeing (Bawden and Robinson, 2009; Spiel et al., 2022). By adopting a proactive mentality, people can transform possible obstacles into chances for development (Caniëls et al., 2018).

Support from friends, family, and the community plays a pivotal role in empowering individuals with ADHD (Craig et al., 2020; Claussen et al., 2022). Educating those around us about the nuances of ADHD fosters understanding (Claussen et al., 2022). Creating an environment that embraces neurodiversity not only reduces stigma but also encourages the celebration of unique perspectives and talents (Armstrong, 2012). In the workplace, accommodations that cater to the specific needs of individuals with ADHD can significantly enhance their performance (Sarkis, 2014). Flexible work arrangements, clear communication channels, and recognition of accomplishments contribute to a positive and inclusive professional environment (Kirby and Smith, 2021). While medication may be a part of the treatment plan for some (Dalton, 2013), it is important to emphasize that there’s no one-size-fits-all solution for managing ADHD (Coghill, 2017). Standard treatment and management for ADHD involve medication, behavioral intervention, nutrition, and supplements is presented in Table 4. Holistic approaches, including mindfulness, exercise, and proper nutrition, can complement traditional treatments and contribute to overall wellbeing (Kobe, 2023). In conclusion, living with ADHD is a journey filled with both challenges and opportunities. By fostering understanding, embracing uniqueness, and developing personalized strategies, individuals with ADHD can navigate life successfully while contributing their distinct talents to the world.

**TABLE 4:** Current attention-deficit hyperactivity disorder (ADHD) treatment plan.

Current treatments	Subgroup	Effects
Medications (FDA, 2023)	- Stimulants, which contains methylphenidate and amphetamine. For example, Intuniv (guanfacine), Strattera (atomoxetine), Kapvay (clonidine) and Qelbree (viloxazine) (FDA, 2023) - Non-stimulants (FDA, 2023)	- Stimulants increases brain levels of dopamine, a neurotransmitter associated with motivation, attention and movement. Increase brain levels of dopamine, a neurotransmitter associated with motivation, attention and movement (FDA, 2023) - Non stimulants medications treat the ADHD symptoms (FDA, 2023)
Cognitive behavioral therapy for affected children (Bradshaw and Kamal, 2017)	Parents and school are provided with behavior-management training programs as well (Al-Ghannami et al., 2018)	Help in managing the symptoms (Bradshaw and Kamal, 2017)
Balanced diet and proper nutrition (Wolraich et al., 2019)	- Specific dietary elimination is advisable if there is a clear link with ADHD behavior (Ferguson, 2000) - Some studies suggested using Omega-3 and Omega-6 fatty acid supplements (Kemper et al., 2018)	There is insufficient evidence on the benefits of using Omega3/6 fatty acid supplements (Kemper et al., 2018)

## 7 Conclusion

The characteristic features of individuals with ADHD include hyperactivity, inattentiveness, and impulsiveness, which lead to impairments in daily living. Research recognized that ADHD was not just a childhood disorder, that disappeared with age as was previously thought. Scientists have been trying to identify the causes of ADHD. It is a clinically and genetically heterogeneous syndrome that is comorbid with childhood conduct disorder, alcoholism, substance abuse, antisocial personality disorder, and affective disorders. The ADHD consistent overlap with autistic symptoms has also been established. Research points to a strong genetic link, yet it is not clear what role environmental factors play in determining who develops ADHD. Psychoeducation, counseling, supportive problem-directed therapy, behavioral intervention, coaching, cognitive remediation, and couples and family therapy are useful adjuncts to medication management.

In conclusion, this comprehensive review underscores the intricate genetic landscape of ADHD. While considerable progress has been made in identifying genetic markers and understanding heritability, continued research efforts are needed to unravel the full complexity of ADHD genetics in different populations, offering hope for more individualized and effective interventions in the future.

## 8 Future perspectives

In the realm of ADHD research in the Arab World, it is imperative to focus on several key aspects for future advancements. First and foremost, there is a critical need to enhance cultural awareness and education surrounding ADHD, dispelling misconceptions, and promoting understanding within the Arab community. Additionally, research efforts should span different demographic levels, exploring variations in ADHD prevalence across urban and rural settings, socioeconomic factors, and access to healthcare resources. Early intervention programs tailored to the unique needs of the Arab population should be established, with training initiatives for educators, parents, and healthcare professionals. Given the cultural landscape, investigating the correlation between consanguinity and ADHD prevalence is crucial, necessitating genetic research to identify specific risk factors. Moreover, GWA and genetic mapping studies should be actively pursued in the Arab World to provide a more holistic insight into the genetic underpinnings of ADHD. Furthermore, community engagement initiatives and support networks can contribute to a more inclusive and understanding environment for individuals affected by ADHD. Finally, improvements in healthcare infrastructure are essential to ensure widespread access to accurate diagnosis and effective treatment, addressing disparities and making ADHD interventions more accessible throughout the Arab World.
